# Unravel the regulatory mechanism of Yrr1p phosphorylation in response to vanillin stress in *Saccharomyces cerevisiae*

**DOI:** 10.1186/s12934-023-02056-8

**Published:** 2023-03-11

**Authors:** Weiquan Zhao, Xinning Wang, Bolun Yang, Ying Wang, Zailu Li, Xiaoming Bao

**Affiliations:** 1 State Key Laboratory of Biobased Material and Green Papermaking, School of Bioengineering, Qilu University of Technology Shandong Academy of Sciences, Jinan, 250353 China; 2 The Second Hospital of Shandong University, Shandong University Library, Jinan, 250100 China

**Keywords:** Yrr1p, Phosphorylation sites, Vanillin resistance, *Saccharomyces cerevisiae*, Subcellular location, Regulation activity

## Abstract

Improving the resistance of *Saccharomyces cerevisiae* to vanillin, derived from lignin, will benefit the design of robust cell factories for lignocellulosic biorefining. The transcription factor Yrr1p mediates *S. cerevisiae* resistance to various compounds. In this study, eleven predicted phosphorylation sites were mutated, among which 4 mutants of Yrr1p, Y134A/E and T185A/E could improve vanillin resistance. Both dephosphorylated and phosphorylated mutations at Yrr1p 134 and 185 gathered in the nucleus regardless of the presence or absence of vanillin. However, the phosphorylated mutant Yrr1p inhibited target gene expression, while dephosphorylated mutants promoted expression. Transcriptomic analysis showed that the dephosphorylated Yrr1p T185 mutant, under vanillin stress, upregulated ribosome biogenesis and rRNA processing. These results demonstrate the mechanism by which Yrr1p phosphorylation regulates the expression of target genes. The identification of key phosphorylation sites in Yrr1p offers novel targets for the rational construction of Yrr1p mutants to improve resistance to other compounds.

**Supplementary Information:**

The online version contains supplementary material available at 10.1186/s12934-023-02056-8.

## Introduction

Eukaryotic cells can resist a range of drugs and toxic compounds, referred to as pleiotropic drug resistance (PDR), by activating pleiotropic membrane transporters located at the plasma membrane and other intracellular membranes [1]. The genes contributing to PDR are primarily ABC transporters, major facilitator superfamily (MFS) transporters, and zinc cluster transcription factors (TFs) [2, 3].

*Saccharomyces cerevisiae* has been a model eukaryotic organism for deciphering genotype–phenotype associations [4]. This organism provides a good foundation for biological refining because of its good fermentation characteristics, high ethanol and low pH tolerance, food-grade biosafety and recognized genetic system. However, *S. cerevisiae* is also limited by the toxicity caused by the accumulation of natural products or by-products [5, 6].

In the biological refining process, vanillin is a typical phenolic inhibitor in the lignocellulosic hydrolysates and interferes with microbial growth and fermentation [7, 8]. Vanillin is one of the most widely used flavors in the food and cosmetics industries thanks to its unique fragrance as well as antimicrobial and antioxidant activities [9—12]. Heterologous biosynthesis of vanillin in *S. cerevisiae* microbial cell factories using a ferulic acid or glucose substrate has become increasingly attractive because the supply of natural vanillin from vanilla pod extracts cannot meet current demand [13—16]. However, vanillin toxicity limits the yield of microbial cell factories [17].

In budding yeast, zinc cluster TFs regulate the PDR network, such as Pdr1p, Pdr3p, Yrm1p, and Yrr1p, orchestrating xenobiotic-induced gene expression [2]. The genetic variation of TFs leads to the differential expression of pleiotropic membrane transporters, producing diverse phenotypes and the ability to adapt to environmental challenges [18, 19]. For example, Ottilie et al. performed in vitro evolution of *S. cerevisiae* to resist diverse compounds and analyzed the whole genomes of the obtained mutants. Resistance to approximately 25% of the compounds was mediated by 100 independent gain-of-function single-nucleotide variations (SNVs) found in a 170-amino acid stretch of Yrr1p and Yrm1p, both Zn_2_C_6_ TFs [20].

*YRR1* was the first zinc cluster transcription factor isolated in an *S. cerevisiae* resistance screen for the cell cycle inhibitor, reveromycin A; its deletion results in hypersensitivity to oligomycin and 4-nitroquinoline-N-oxide (4-NQO) [21]. Yrr1p binds to the promoters of *YOR1* and *SNQ2,* which encode PDR ABC transporters and mediate resistance to hundreds of drugs along with the other ABC transporter Pdr5p [22]. Yrr1p also regulates the MFS transporters *AZR1*, *SNG1*, and *FLR1* [23]. Kodo et al. found a novel *YRR1* named *YRR1-52*, which conferred increased resistance to salicylic acid by activating the expression of efflux pump-encoding genes *YOR1*, *SNQ2*, *AZR1*, and *FLR1* [22]. Teixeira et al. found that yeast adaptation to Mancozeb involves the upregulation of *FLR1* under the coordinated control of Yrr1p, Yap1p, Rpn4p, and Pdr3p [24]. Gallagher et al. found that Yrr1p^YJM789^, which confers 4-NQO resistance, is associated with T775 and S759 phosphorylation [25]. In our previous study, *YRR1* in BY4741 showed no remarkable change in mRNA and protein content in response to vanillin stress, while its target *SNQ2* was upregulated [26]. Therefore, vanillin may upregulate downstream target expression by regulating the activity of Yrr1p rather than its expression. The change in transcription factor regulatory activity probably depends on the modification in phosphorylation [27]. Herein, the potential phosphorylation sites of Yrr1p were systematically analyzed and mutated to glutamate or alanine. We focused on the regulation and subcellular localization of Yrr1p as a result of phosphorylation site mutations.

## Result

### Translocation of Yrr1p in response to vanillin stress

**Fig. 1:**
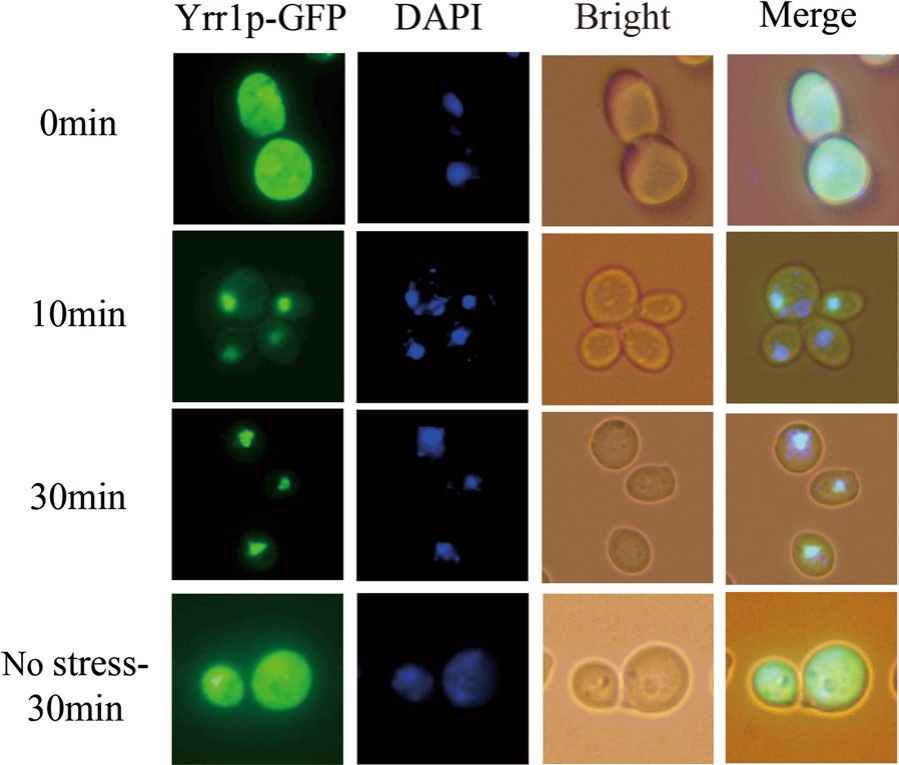
Subcellular localization of Yrr1p in vanillin environment. The cells were exposed to 6 mM vanillin stress with 0, 10 and 30 min in SC-Ura medium. The localization of Yrr1p GFP was analyzed by fluorescence microscope (green). Nuclear DNA was stained with DAPI (blue)

To avoid the occupancy effect of in situ Yrr1p on chromosomes, we chose to express Yrr1p carrying a carby-terminal green fluorescent protein (GFP) in BY4741(*yrr1Δ*). *YRR1*-GFP was cloned into centromeric plasmid pJFE1 under the control of the TEF1 promoter and PGK1 terminator. The recombinant plasmid was transformed into BY4741(*yrr1Δ*) to obtain recombinant strain BY4741(*yrr1Δ* + *YRR1*-GFP), and the time-dependent subcellular location of Yrr1p was detected by fluorescence microscopy after exposure to 6 mM vanillin. As shown in Fig. 1, in the absence of vanillin, Yrr1p-GFP was distributed throughout the cell. When cells were exposed to 6 mM vanillin for 10 min, most of the Yrr1p-GFP translocated from the cytoplasm to the nucleus.

**Fig. 2:**
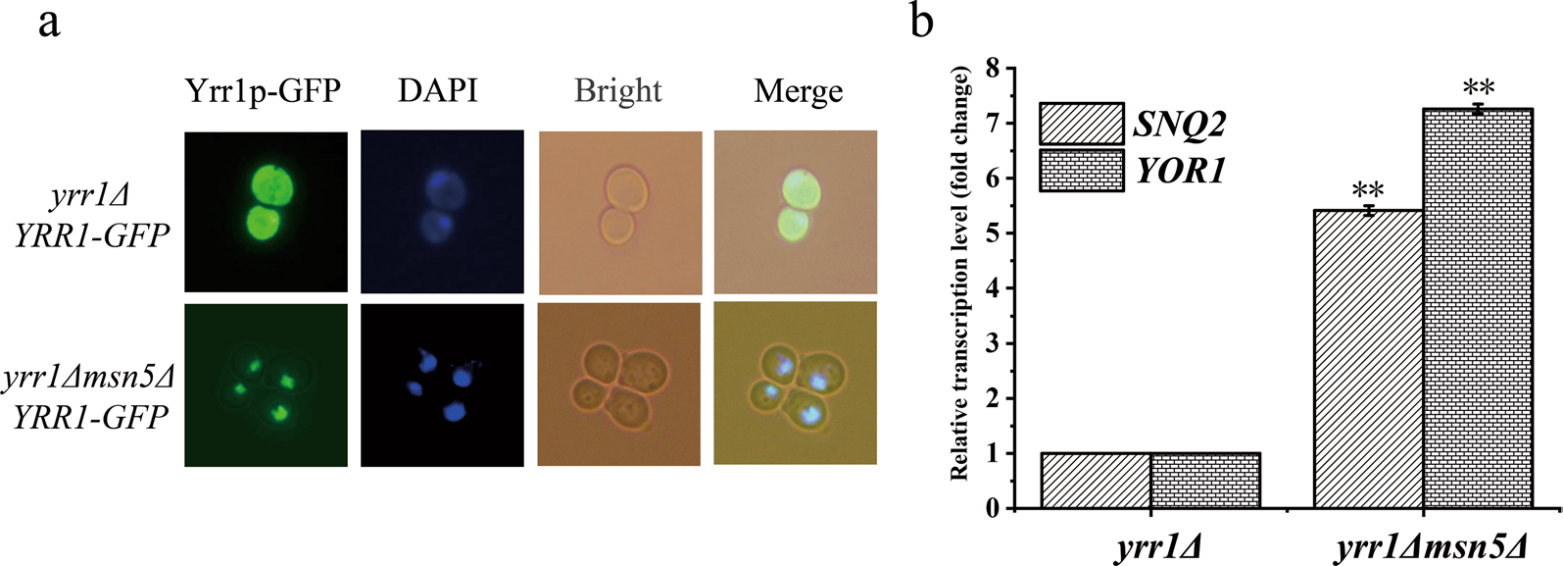
The localization of Yrr1p affected by *MSN5* (**a**). Fluorescence microscope (green) showing Yrr1p-GFP in SC-Ura medium. Nuclear DNA was stained with DAPI (blue). The transcription level of the *SNQ2* and *YOR1* genes relative to reference gene *ACT1* (**b**). The error bar displays the standard deviation of triplicates. Significance level *: *p-value* < 0.05, **: *p-value* < 0.01

Exportin-5 is an evolutionarily conserved nuclear export factor of the nuclear protein family that exports phosphorylated proteins and small noncoding RNAs [28, 29]. *MSN5* is the homologue of Exportin-5 and exports a large number of phosphorylated cargoes: endonuclease, cyclin-dependent kinase inhibitor Far1p, transcription factors Crz1p, Pho4p, Mig1p and Aft1p, scanning protein Step5p, chaperones Hsp70p and Ssa4p, and RNA polymerase III regulatory protein Maf1p [30]. After *MSN5* deletion in BY4741(*yrr1Δ* + *YRR1*-GFP), Yrr1p aggregated in the nucleus even in the absence of vanillin stress (Fig. 2a). The mRNA content of *SNQ2* and *YOR1*, which were activated by Yrr1p, was also upregulated by 4.4- and 6.3-fold, respectively, by knockdown of *MSN5* (Fig. 2b). Overexpression of *SNQ2* and *YOR1* improves vanillin resistance [26]. These findings suggest that Yrr1p is dephosphorylated in response to vanillin stress, migrating into the nucleus to activate the expression of downstream target genes. The absence of *MSN5* leads to constitutive activation of Yrr1p targets in the presence or absence of vanillin stress. However, instead of improving vanillin resistance, the deletion of *MSN5* inhibit the growth of *S. cerevisiae* (Additional file 1: Figure S2). This was probably due to the accumulation of multiple phosphorylated protein in the cell nucleus and result in growth burden.

### Bioinformatics analysis of Yrr1 phosphorylation sites

**Fig. 3:**
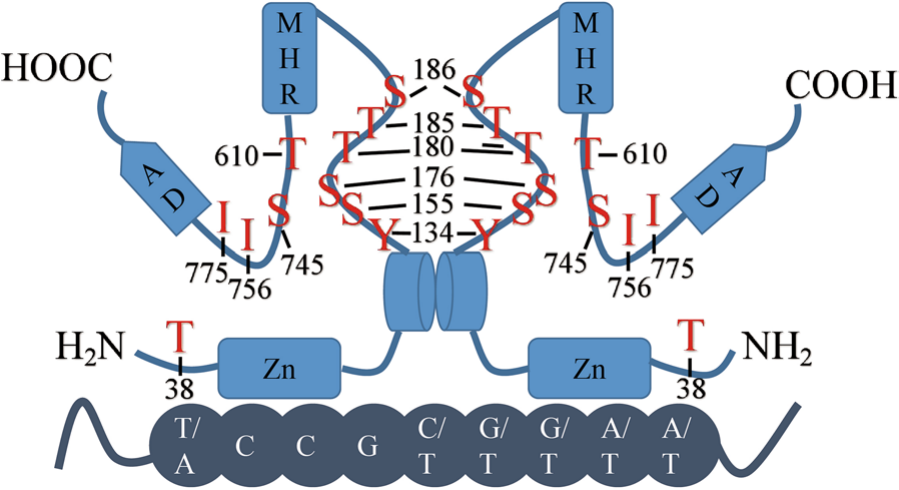
Domain and position of the 11 predicted phosphorylation sites of Yrr1p. (*Zn* Zn finger-binding domain, *MHR* middle homology region, *AD* activation domains)

*YRR1* is located on chromosome XV of *S. cerevisiae*, and its full length is 2466 bp, encoding the 810-amino acids of Yrr1p protein. From the amino terminus to the carboxy terminus, Yrr1p contains each of Zn finger-binding domain (Zn), middle homology region (MHR), and activation domains (AD) (Fig. 3). There is a zinc finger motif of Zn(II)2Cys6 type in the DNA binding domain, and a short α coiled-coil exists around the binding domain, mainly involved in the homodimer reaction of *YRR1* [3, 31]. Each domain performs different functions [23]. PhosphoSitePlus, iGPS, Uniprot, and DTU Health Tech were used to analyze potential Yrr1p phosphorylation sites. Nine possible phosphorylation sites were predicted (T38, Y134, S155, S176, T180, T185, S186, T610, and S745). Gallagher et al. found 775E, 775A, 756E, and 756A mutations in the two alleles of *YRR1* when screening the 4-NQO resistant strain of *S. cerevisiae* and speculated that they were possible phosphorylation sites, so we also took these two sites as candidates (I756 and I775) [25]. The positions of potential phosphorylation sites in Yrr1p are shown in Fig. 3.

Of the 11 possible phosphorylation sites, none were distributed in the three domains of *YRR1*. There are six predicted phosphorylation sites (Y134, S155, S176, T180, T185, and S186) between the DNA-binding domain and the central regulatory domain α-helical structure, and four predicted phosphorylation sites (T610, S745, I756, and I775) between the central regulatory and active domains. The predicted phosphorylation site T38 is located near the N-terminal side of the DNA-binding domain.

### Effects of single-site Yrr1p dephosphorylation mutations on function

**Fig. 4:**
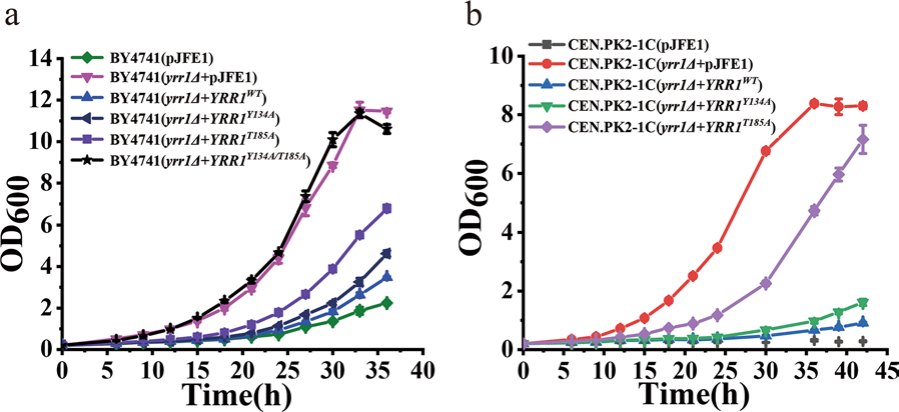
The vanillin resistance of dephosphorylation mutation strains. The recombinant strains with different host strains BY4741 (**a**) and CEN.PK2-1C (**b**) were incubate in SC-Ura medium supplemented with 6 mM vanillin at 30 ℃. The error bar represents the standard deviation of triplicates

Natural polymorphisms in a single regulatory gene can lead to a wide range of molecular changes and individual phenotypes [32]. Serine, threonine, and tyrosine are negatively charged after phosphorylation. These 11 sites were mutated to uncharged alanine to simulate the intracellular dephosphorylation state. The 11 *YRR1* mutations were cloned into pJFE1, and the recombinant plasmids were converted into BY4741(*yrr1Δ*) to eliminate the occupancy effect of in situ *YRR1*. Fermentation showed that under 6 mM vanillin stress, the maximum specific growth rates of *YRR1*^*Y134A*^ and *YRR1*^*T185A*^ dephosphorylation mutants were 9.6–34.4% higher than the BY4741(*yrr1Δ* + *YRR1*^*WT*^), and the lag phases were shortened by 2 and 5 h, respectively (Figs. 4a and Additional file 1: Fig. S1). The lag phases of the corresponding mutants were reduced to 22 and 19 h. The other nine unit point mutations could not improve the vanillin tolerance of *S. cerevisiae*. Therefore, positions 134 and 185 are crucial sites for Yrr1p-mediated vanillin resistance. Under 6 mM vanillin stress, the maximum specific growth rate of the 134/185 double mutation strain BY4741(*yrr1Δ* + *YRR1*^*Y134A/T185A*^) was 27.7%, 4.2%, and 40.0% higher than the 134 and 185 single mutations and BY4741(*yrr1Δ* + *YRR1*^*WT*^), respectively. The lag phase of the double dephosphorylation mutation was shortened by 12 h compared to BY4741(*yrr1Δ* + *YRR1*^*WT*^) (Table 1). The two dephosphorylation mutations also increased the vanillin resistance of CEN.PK2-1C, whose genetic background was very different from that of BY4741 (Fig. 4b). Under 6 mM vanillin stress, the maximum specific growth rates of the 134 and 185 mutants were increased by 143.3% and 263.3%, respectively (Table 1).

**Table 1:** Maximum specific growth rate and lag phase of dephosphorylation mutants under 6 mM vanillin stress

Strains	SC-Ura with 6 mM vanillin	
	μ_max_ (h^−1^)	Lag phase(h)
*yrr1Δ* + *YRR1*^*WT*^	0.125 ± 0.003	24
*yrr1Δ* + *YRR1*^*Y134A*^	0.137 ± 0.003^**^	22
*yrr1Δ* + *YRR1*^*T185A*^	0.168 ± 0.002^*^	19
*yrr1Δ* + *YRR1*^*Y134A/T185A*^	0.175 ± 0.002^*^	12
CEN.PK2-1C (*yrr1Δ* + *YRR1*^*WT*^)	0.030 ± 0.001	> 42
CEN.PK2-1C (*yrr1Δ* + *YRR1*^*Y134A*^)	0.073 ± 0.003^*^	36
CEN.PK2-1C (*yrr1Δ* + *YRR1*^*T185A*^)	0.109 ± 0.004^*^	22

^*^ *p*-value < 0.001; ** *p*-value < 0.01

**Fig. 5:**
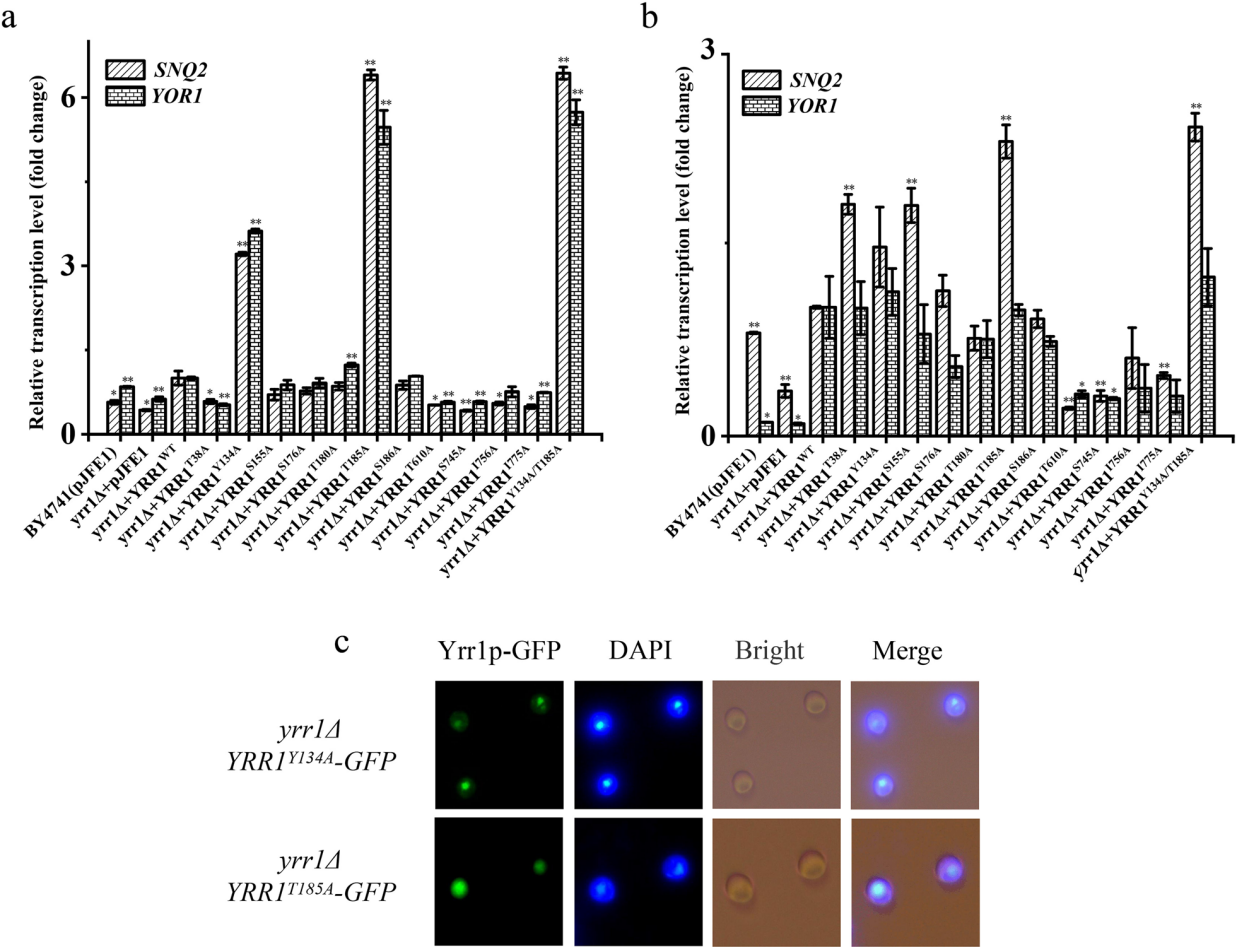
The mRNA of the target genes in *YRR1* dephosphorylation mutations in SC-Ura medium (**a**) and under 6 mM vanillin stress (**b**). The error bar represents an independent 3 times the standard deviation (**c**). The subcellular localization of dephosphorylation mutations under vanillin-free condition. The intracellular localization (green) was analyzed by fluorescence microscope. Nuclear DNA was stained with DAPI (blue). Significance level *: *p-value* < 0.05, **: *p-value* < 0.01

RT-PCR showed that in the absence of vanillin stress, the relative expression of the target genes in *YRR1*^*T134A*^ and *YRR1*^*T185A*^ mutants was upregulated by 2.2- and 5.4-fold (*SNQ2*) and 2.6- and 4.5-fold (*YOR1*), respectively, compared to the BY4741(*yrr1Δ* + *YRR1*^*WT*^). Transcript levels of *SNQ2* and *YOR1* in the double mutation strain were 6.4- and 5.7-fold greater than in BY4741(*yrr1Δ* + *YRR1*^*WT*^) (Fig. 5a). Under 6 mM vanillin stress, the relative expression of target genes *SNQ2* in the presence of mutations at 38, 134, 155, and 185 were 1.8-, 1.5-, 1.8-, and 2.3-fold greater than BY4741(*yrr1Δ* + *YRR1*^*WT*^), respectively (Fig. 5b).

Under stress-free conditions, fluorescence microscopy showed that Yrr1p^*Y134A*^ and Yrr1p^*T185A*^ aggregate in the nucleus (Fig. 5c), indicating that the dephosphorylated mutations at positions 134 and 185 likely caused Yrr1p to be anchored in the nucleus and continuously activate the expression of *SNQ2* and *YOR1* in the absence of vanillin. *SNQ2* and *YOR1*, encoding ABC transporters, was reported participate in the efflux of many drugs [33, 34]. Our previous work also proved that the overexpression of these two genes can shorten the delay period and improve the vanillin tolerance of *S. cerevisiae* [26]. Dephosphorylated Yrr1p can accumulate in the nucleus even without vanillin stress, so that *SNQ2* and *YOR1* have a high level of expression. Thus, when vanillin was added, *SNQ2* and *YOR1* probably react immediately to excreted vanillin out of cells, thereby shortening the lag phase and enhancing the vanillin resistance of *S. cerevisiae.*

### Effects of single-site phosphorylation mutations of Yrr1p on its function

**Fig. 6:**
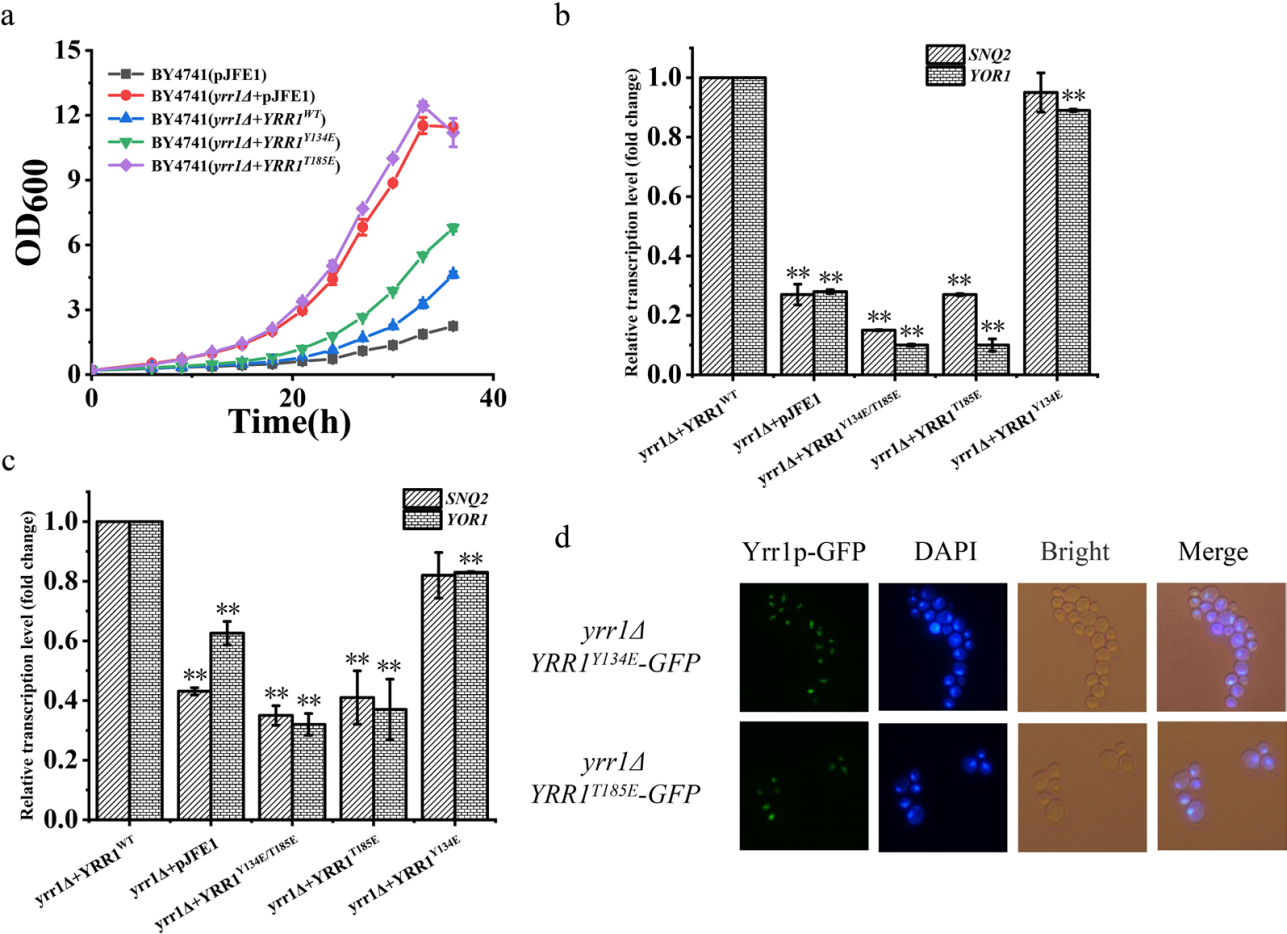
The vanillin resistance profiling of phosphorylation mutations strians cultured in SC-Ura with 6 mM vanillin stress (**a**). The relative expression of the target genes of phosphorylation mutants in SC-Ura medium with 6 mM vanillin (**b**) and without stress(**c**). The error bar represents an independent 3 times the standard deviation. Subcellular localization of phosphorylation mutations without vanillin stress (**d**). Intracellular localization was analyzed by fluorescence microscope (green). Nuclear DNA was stained with DAPI (blue). Significance level *: *p-value* < 0.05, **: *p-value* < 0.01

Amino acids 134 and 185 were mutated to glutamic acid to mimic the intracellular phosphorylation state. The fermentation results show that, compared to BY4741(*yrr1Δ* + *YRR1*^*WT*^), the maximum specific growth rates of BY4741(*yrr1Δ* + *YRR1*^*Y134E*^) and BY4741(*yrr1Δ* + *YRR1*^*T185E*^) increased by 12.8–20.5% under 6 mM vanillin stress, respectively (Fig. 6a and Table 2). Transcript levels of *SNQ2* and *YOR1* in the *YRR1*^*Y134E*^, *YRR1*^*T185E*^, and *YRR1*^*Y134E/T185E*^ phosphorylation mutants decreased by 4.6%, 73.3%, and 85.2% for *SNQ2* and by 11.1%, 90.0%, and 90.0% for *YOR1*, respectively (Fig. 6b). Target gene expression levels in phosphorylated mutant strains under vanillin-free conditions also reduced, 18.2%, 59.3%, and 65.5% for *SNQ2* and 17.5%, 63.5%, and 68.2% for *YOR1* compared with the BY4741(*yrr1Δ* + *YRR1*^*WT*^) (Fig. 6c). Therefore, phosphorylation of Yrr1p at positions 134 and 185 inhibited target gene expression. The phosphorylated mutants of Yrr1p remained localized to the nucleus in the presence or absence of vanillin (Fig. 6d).

**Table 2:** Maximum specific growth rate and lag phase of the phosphorylation mutants under 6 mM vanillin stress

Strains	SC-Ura with 6 mM vanillin	
	μ_max_ (h^−1^)	Lag phase(h)
*yrr1Δ* + *YRR1*^*WT*^	0.117 ± 0.001	26
*yrr1Δ* + *YRR1*^*Y134E*^	0.132 ± 0.002^*^	23
*yrr1Δ* + *YRR1*^*T185E*^	0.141 ± 0.002^*^	12

^*^ *p*-value < 0.001

### Transcriptome analysis of Yrr1p regulatory activity with and without phosphorylation at position 185

**Fig. 7:**
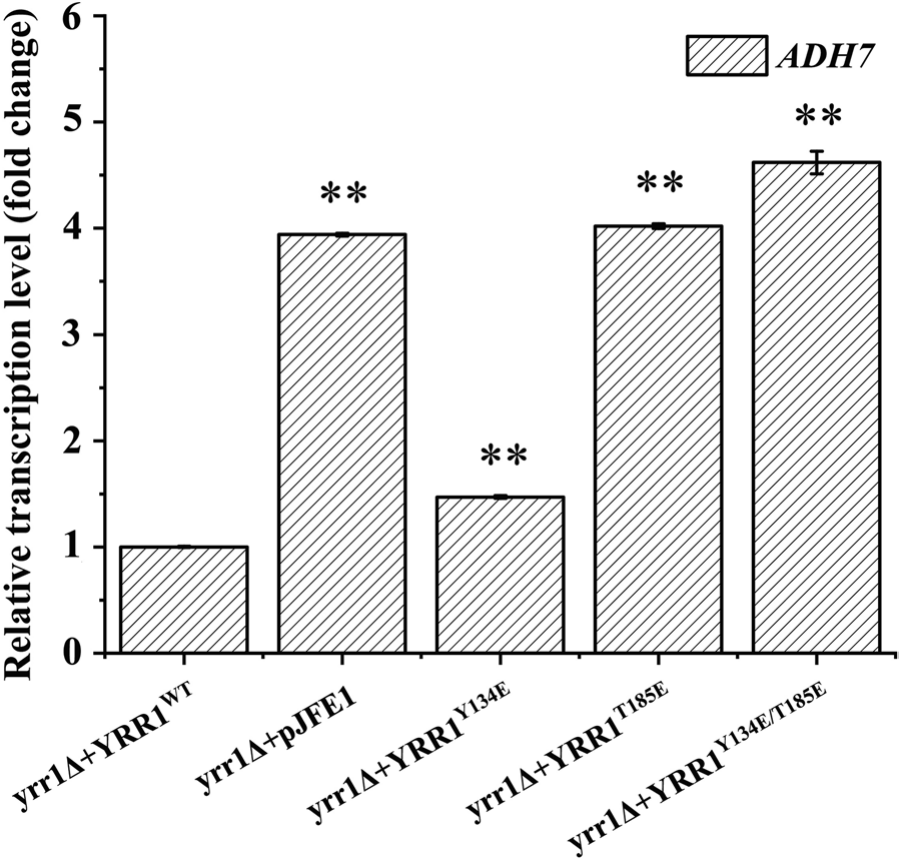
Relative expression of target gene *ADH7* in phosphorylation mutants in SC-Ura medium. The error bar represents 3 times the standard deviation. Significance level *: *p-value* < 0.05, **: *p-value* < 0.01

To investigate the differences in global mRNA expression in the presence of phosphorylated and dephosphorylated Yrr1p, we performed transcriptome analyses of BY4741(*yrr1Δ* + *YRR1*^*T185A*^), BY4741(*yrr1Δ* + *YRR1*^*T185E*^), and BY4741(*yrr1Δ* + *YRR1*^*WT*^) in SC-Ura in the presence and absence of 6 mM vanillin. Genes exhibiting remarkable differential expression are listed in Table 3. Compared to the wild-type *YRR1-*compensated strain, 75 genes related to ribosome biogenesis and rRNA processing were upregulated in BY4741(*yrr1Δ* + *YRR1*^*T185E*^). The dehydrogenase gene *ADH7* was upregulated. The pattern of mRNA expression in BY4741(*yrr1Δ* + *YRR1*^*T185E*^) was similar to the *YRR1*-deletion strain. Deleting *YRR1* also increased the expression of *ADH7* and genes related to ribosome biogenesis and rRNA processing in the presence of vanillin [26]. RT-PCR confirmed that under 6 mM vanillin stress, *ADH7* expression in the Yrr1p 134/185 single- or double-site phosphorylation mutants was upregulated by 0.5-, 3.0-, and 3.6-fold (Fig. 7). Adh7p and its homologous gene, Adh6p were confirmed to convert vanillin to vanillyl alcohol, which is less toxic than vanillin. The overexpression of *ADH7* and *ADH6* was confirmed to remarkably improve vanillin resistance [26, 35, 36].

**Table 3:** The vanillin response genes and the genes significantly regulated by Yrr1p with vanillin stress

Function	BY4741(*yrr1Δ* + *YRR1*^*T185A*^) *vs* BY4741(*yrr1Δ* + *YRR1*^*WT*^)	BY4741(*yrr1Δ* + *YRR1*^*T185E*^) *vs* BY4741(*yrr1Δ* + *YRR1*^*WT*^)	BY4741(*yrr1Δ* + *YRR1*^*T185E*^) *vs* BY4741(*yrr1Δ* + *YRR1*^*T185A*^)
Ribosome biogenesis and rRNA processing	*DBP2, NSR1*	*ARPS16A, RPL34A, RPP1A, RPL1A, RPL4A, RPS19A, RPL19A, RPL38, RPS10B, RPL4B, RPL36B, RPL31A, RLP24, RPL40B, RPL33A, RPL19B, DRS1, RPL32, TSR1, RPS18B, RPL27B, RPL23A, RPL12B, DBP9, RPS22B, RPL20B, RPL13A, RPS10A, RPL7A, RPL22A, RPA34, RPL11A, RPL24A, RPL37A, RPL26B, RPL16A, RPL13B, RPS24B, RPS11A, DBP3, RPS9B, RPS11B, NMD3, RPA12, RPL17B, DBP8, RPS16B, RPS1A, RPL16B, RPL20A, RPL31B, RPL11B, RPL15A, PRP43, RPL6B, RPS8B, RPS17B, MTR4, RPL1B, RPL2A, RPS31, RPA190, RPL8B, NOG1, RPL21A, RPL9A, RPA49, RPS0B, RPA43, RPA135, RPS26B, RPS0A, HAS1, NSR1, DBP2*	*MAK1, RPS26B, BRX1, DBP2*
Dehydrogenase	None	*GND2, AAD4, DSF1, BDH1, IDP2, ALD3, ARA1, SHH4, SHH4, GRE3, GCY1, SDH3, ETR1, BDH2, MDH1, HFD1, MDH2, SDH1, SDH8, SDH5, UGA2* *AAD6, HOM2, HIS4, MIS1, IMD4, DLD3, ADH7, LYS9, LYS12, ALD5*	*GND2, AAD4, IDP2, BDH1, DSF1, ARA1, ALD3*
ATP-binding cassette (ABC) transporter	None	*SNQ2, YOR1, PDR15* *NEW1, PDR5*	*SNQ2, YOR1*

Genes that are underlined are down-regulated; the others are up-regulated

The genes listed in the table are those for which log2 > 1, *p*-value < 0.05

## Discussion

Natural polymorphisms in a single important transcriptional factor result in distinct regulatory programs and phenotypes in individuals. Yrr1p was found to play an important role in the evolution of multidrug resistance [20]. Gallagher et al. found that the phenotypic difference was largely associated with changes in a single amino acid in Yrr1p that was a potential site of phosphorylation. In this study, we systematically analyzed the potential phosphorylation sites of Yrr1p and mutated each site to assess its effect on the regulatory activities of Yrr1p and resistance to vanillin, an important phenolic inhibitor of lignocellulosic hydrolysates.

In the absence of vanillin stress, Yrr1p is localized to the cytoplasm. In response to vanillin stress, Yrr1p translocates from the cytoplasm to the nucleus, where it induces the expression of *SNQ2* and *YOR1*, which encode ABC transporters. Deletion of the exportin gene *MSN5* led to Yrr1p accumulation in the nucleus even in the absence of vanillin. The nuclear accumulation of Yrr1p induced the expression of its target genes, *SNQ2* and *YOR1*, without vanillin stress. As Msn5p is known to preferentially export the phosphorylated forms of transcriptional factors from the nucleus, these results indicated that Yrr1p is phosphorylated in the absence of stress and dephosphorylated in response to vanillin stress. The phosphorylation status of Yrr1p affected its subcellular localization and the expression of target genes.

Among the 11 sites of potential phosphorylation screened by bioinformatics, amino acids 134 and 185 were the crucial sites for Yrr1p-mediated vanillin resistance. The phosphorylation or dephosphorylation of these sites led to an obvious improvement in resistance to vanillin stress. Amino acid 185 was the more dominant site, as the increase of vanillin resistance and upregulation of target gene expression were most remarkable with the mutation of this residue.

The dephosphorylation of Yrr1p located in cell nucleus because Msn5p only exported the phosphorylated protein out of nucleus. To our surprise, phosphorylated mutant Yrr1p can still accumulate in the nucleus. There may be a nuclear export sequence (NES) disrupted by mutation at these two sites. However, no conserved NES has been identified in a large variety of proteins that depend on Msn5 for nuclear export [29]. Phosphorylation at residues 134 and 185 was speculated to prevent Yrr1p from binding to its target gene promoters, as the two sites are close to the DNA-binding domain of Yrr1p.

The mechanism toward improving vanillin resistance of phosphorylation and dephosphorylation mutations were different. The dephosphorylation mutation was anchored in the nucleus and made continuous expression of *SNQ2* and *YOR1,* whose overexpression were proved to shortening the lag phase and enhancing the vanillin resistance of *S. cerevisiae* in our previous work. [26] The transcriptome analysis showed that in response to vanillin stress, the phosphorylated mutant Yrr1p strain had similar patterns of mRNA expression with the *YRR1* deletion strain. Vanillin was reported to inhibit ribosome biogenesis [37]. The ribosome proteins in BY4741 were decreased by vanillin stress [38]. Both the *YRR1* deletion strain and the phosphorylated mutant released this inhibition [26, 38]. The ribosomal related genes and rRNA processing‑related genes including the ATP-dependent RNA helicase *DBP2,* whose overexpression was proved enhancing vanillin resistance, [26] were significantly upregulated in phosphorylated mutant. Besides, the expression of alcohol dehydrogenase *ADH7* was also increased in both phosphorylated mutant and *YRR1* deletion strain, which accelerates the reduction of vanillin to low-toxic vanillyl alcohol.

Phosphorylation/dephosphorylation at Yrr1p positions 134 and 185 had no effect on resistance to furfural and 5-hydroxymethylfurfural (HMF), which are furan inhibitors of lignocellulosic hydrolysate (Additional file 1: Fig S3a–c). This result showed that the enhanced resistance caused by the phosphorylation mutation of *YRR1* was vanillin-specific. In addition, the 4-NQO resistance of the strain has been proved to be related to the expression of *SNQ2*, [21, 39, 40] and the phosphorylation of these positions has indeed improved resistance to 4-NQO, a carcinogenic inducer (Additional file 1: Fig S4).

## Materials and methods

### Culture media

Yeast extract peptone dextrose (YEPD) medium (10 g L^−1^ yeast extract, 20 g L^−1^ tryptone, and 20 g L^−1^ glucose) was used for activation, culture of host strains, and spot dilution growth (with 2% agar).

SD or SC-Ura medium (1.77 g L^−1^ yeast nitrogen base, 5 g L^−1^ ammonium sulfate, 0.77 g L^−1^ CSM or CSM-URA) was used for activation and batch fermentation of recombinant strains, supplying 20 g L^−1^ glucose. Vanillin was used as an inhibitor and added to the medium as indicated. All cultures were grown at 30 °C unless otherwise indicated.

### Strains and plasmids

Genetic manipulations, including deletion and overexpression, were primarily conducted in the lab strains BY4741 (*MATa; his3Δ1, leu2Δ, met5Δ,* and *ura3Δ;* EUROSCARF, Germany) and CEN.PK2-1C (*MATa; ura3-52; trp1-289; leu2-3,112; his3Δ 1; MAL2-8C; SUC2,* EUROSCARF). The two strains have different genetic backgrounds. Gene deletion was conducted using homologous recombination [41]. The *YRR1*-deleted strain CEN.PK2-1C(*yrr1Δ*) (CEN.PK2-1C derivative; *yrr1::loxP*), BY4741(*yrr1Δ*) (BY4741 derivative; *yrr1::loxP*) and *YRR1-*compensated strain BY4741(*yrr1Δ*, *YRR1*^*WT*^) (BY4741 derivative; *yrr1::loxP*, pJFE1-*YRR1*^*WT*^) were previously stored and constructed in our laboratory. The destruction cassette contained sequences homologous to the genes for knockout and the *KanMX* expression cassette amplified from plasmid pUG6 [42]. Destruction cassettes were then transformed into BY4741, and the mutants were screened in YEPD medium containing 800 mg mL^−1^ G418.

Laboratory-developed plasmid pJFE1 is a centromere plasmid with the *TEF1* promoter, *PGK1* terminator, and *URA3* expression cassette as the selection marker [26]. The *YRR1* gene was amplified from the genomic DNA of BY4741 and inserted into pJFE1. The single- and double-point mutations of *YRR1* were designed in the primer sequence and obtained by overlap extension PCR. To observe the subcellular localization of Yrr1p, the mutant and *YRR1* were fused with GFP using overlap extension PCR. The PCR products were digested with *Bam*HI and *Pst*I and then cloned into predigested pJFE1. All the strains and primers used in this study are listed in Additional file 1: Table S1.

### Bioinformatics analysis

The phosphorylation site analysis tools Pfamscan, iPTMnet, and DTU.dk were used to analyze Yrr1p phosphorylation; combined with literature reports, we predicted 11 potential phosphorylation sites and established a Yrr1p phosphorylation site database. The candidate positions are 38, 134, 155, 176, 180, 185, 186, 610, 745, 756, and 775. These sites were mutated to alanine to mimic dephosphorylation status or to glutamate to mimic phosphorylation status.

### Fluorescence microscopy

The Yrr1p-mutated yeast cells were grown in 40 mL SC-Ura medium, cultured at 30 ℃ to the logarithmic growth stage (optical density at 600 nm [OD_600_] = 1.0). Then the cultures were subjected to vanillin stress by adding vanillin to a final concentration of 6 mM. After 0, 10, and 30 min of cultivation, cells were fixed (0.1 M sorbitol, 40% absolute ethanol) for 5 min. DAPI (4ʹ, 6-diamidino-2-phenylindole, a fluorescent dye capable of strongly binding to DNA) was added to a final concentration of 50 ng mL^−1^, and the cells were observed under a fluorescence microscope (Nikon MODEL ECLIPSE CI-L) with the following parameters: ex 361–389; DM: 415; BA: 430–490; FITC: EX465–495; DM: 505; BA: 512–558 [43].

### Profiling of vanillin resistance

The cells were cultured overnight after being precultured for 24-h in 10 mL fresh medium with an initial OD_600_ of 0.2. The overnight culture was batched in 40 mL of fresh SC-Ura with 6 mM vanillin. Fermentation was carried out at 30 ℃ and 200 rpm. The maximum growth rates were regarded as the linear regression coefficients of the ln (OD_600_) versus time during the exponential growth phase [38].

### Quantitative real-time PCR

Yeast cells were grown in 40 mL SC-Ura medium at 30 ℃ to logarithmic growth phase at 1.0 of OD_600_, then incubated with or without 6 mM vanillin stress for 60 min. Total RNA was isolated using the UNIQ-10 Column Trizol Total RNA Isolation Kit (Sangon Biotech, China) according to manufacturer instructions. After the removal of gDNA, the ABScript II RT Master Mix reverse transcription kit (PK20403/Wuhan Ebotec Biotechnology Co., Ltd.) was used to reverse transcribe the processed total RNA into cDNA. Quantitative PCR reactions were performed with the SYBR Green Master Mix Kit (Wuhan Aibotaike Biotechnology). Transcript levels were normalized to *ACT1*. Primers are listed in Additional file 1: Table S2.

### Transcriptome analysis

The precultured BY4741(*yrr1Δ* + *YRR1*^*T185A*^), BY4741(*yrr1Δ* + *YRR1*^*T185E*^), and BY4741(*yrr1Δ* + *YRR1*^*WT*^) in SC-Ura medium were transferred to a fresh SC-Ura medium with an initial OD_600_ of 0.2. At OD_600_ of 1.0, the cells were induced with or without 6 mM vanillin stress for 1 h, then quick-frozen in liquid nitrogen. Total RNA was extracted using a UNIQ-10 Trizol RNA Purification Kit (Sangon Biotech, China); then, mRNA was isolated, fragmented, and used as a template for cDNA. The short fragments were joined with adapters to obtain suitable fragments for PCR amplification. The libraries were sequenced on an Illumina HiSeq™ 4000 (BGI Shenzhen, China). Differentially expressed genes were screened according to the following criteria: log2 ≥ 1 and P-value < 0.05. All analyses were performed in biological triplicate. Transcriptome data were deposited in the NCBI Sequence Read Archive (accession number: PRJNA878623). Gene functions were annotated using the tool on *Saccharomyces genome database*.

## Supplementary Information

**Additional file 1**: **Table S1.** Yeast strains used in this study. **Table S2.** List of primers used for plasmids and strain construction in this work. **Figure S1. **Growth curve of all eleven point mutants under 6 mM vanillin stress in SC-Ura medium. The error bar represents three times the standard deviation. **Figure S2.** Resistance test of recombinant strains. The host strains were all BY4741. Incubate in SC-Ura liquid medium supplemented with 12 mM furfural (a), 20 mM HMF (b) and no inhibitor (c) at 30℃. The error bar represents three times the standard deviation. **Figure S3.** Resistance test of recombinant strains. The host strains were all BY4741. Incubate in SC-Ura liquid medium supplemented with 0.05 mg L^-1^ 4NQO at 30℃. The error bar represents three times the standard deviation. **Figure S4.** Subcellular localization of two site phosphorylation and dephosphorylation mutations. The samples were cultured in SC-Ura. Intracellular localization was analyzed by fluorescence microscope (green). Nuclear DNA was stained with DAPI (blue). **Figure S5.** Subcellular localization of two site phosphorylation and dephosphorylation mutations. The samples were cultured in SC-Ura. Intracellular localization was analyzed by fluorescence microscope (green). Nuclear DNA was stained with DAPI (blue).
