# Supplementary material for: Unravel the regulatory mechanism of Yrr1p phosphorylation in response to vanillin stress in Saccharomyces cerevisiae
Source: Microb Cell Fact. 2023 Mar 11;22:48. doi: 10.1186/s12934-023-02056-8 (PMC10007725; doi:10.1186/s12934-023-02056-8)
Supplement: Supplementary file 1 — Additional file 1: Table S1. Yeast strains used in this study. Table S2. List of primers used for plasmids and strain construction in this work. Figure S1. Growth curve of all eleven point mutants under 6 mM vanillin stress in SC-Ura medium. The error bar represents three times the standard deviation. Figure S2. Resistance test of recombinant strains. The host strains were all BY4741. Incubate in SC-Ura liquid medium supplemented with 12 mM furfural (a), 20 mM HMF (b) and no inhibitor (c) at 30℃. The error bar represents three times the standard deviation. Figure S3. Resistance test of recombinant strains. The host strains were all BY4741. Incubate in SC-Ura liquid medium supplemented with 0.05 mg L-1 4NQO at 30℃. The error bar represents three times the standard deviation. Figure S4. Subcellular localization of two site phosphorylation and dephosphorylation mutations. The samples were cultured in SC-Ura. Intracellular localization was analyzed by fluorescence microscope (green). Nuclear DNA was stained with DAPI (blue). Figure S5. Subcellular localization of two site phosphorylation and dephosphorylation mutations. The samples were cultured in SC-Ura. Intracellular localization was analyzed by fluorescence microscope (green). Nuclear DNA was stained with DAPI (blue). [file 12934_2023_2056_MOESM1_ESM.docx]

# Additional file 1

**Unravel the regulatory mechanism of Yrr1p phosphorylation in response to vanillin stress in *Saccharomyces cerevisiae***

Weiquan Zhao^1^, Xinning Wang^1,2^*, Bolun Yang, Ying Wang, Zailu Li, Xiaoming Bao*

^1^State Key Laboratory of Biobased Material and Green Papermaking, School of Bioengineering, Qilu University of Technology (Shandong Academy of Sciences), Jinan 250353, China

^2^Shandong University Library, Jinan 250100, China

**Table S1.** Yeast strains used in this study

| Strains | Properties | Source/reference |
| --- | --- | --- |
| BY4741 | *S. cerevisiae* laboratory strain, MATα his3-∆1 leu2-∆0 met15-∆0 ura3-∆0 | Euroscarf |
| BY4741(*yrr1Δ*) | BY4741 derivate; *yrr1*::loxP | Lab preservation |
| BY4741(*yrr1Δmsn5Δ*) | BY4741 derivate; *yrr1::loxP, msn5::loxP-KanMX4- loxP* | This work |
| BY4741(*yrr1Δ+YRR1-*GFP) | BY4741 derivate; *yrr1::loxP*; pJFE3-*YRR1-*GFP | This work |
| BY4741(*yrr1Δmsn5Δ+YRR1-*GFP) | BY4741 derivate; *yrr1::loxP, msn5::loxP-KanMX4- loxP*; pJFE3-*YRR1-*GFP | This work |
| BY4741(*yrr1Δmsn5Δ+YRR1*) | BY4741 derivate; *yrr1::loxP, msn5::loxP-KanMX4- loxP*; pJFE1-*YRR1* | This work |
| BY4741(pJFE1) | BY4741 derivate; pJFE1 | This work |
| BY4741(*yrr1Δ+*pJFE1) | BY4741 derivate; *yrr1::loxP*; pJFE1 | This work |
| BY4741(*yrr1Δ+YRR1^WT^*) | BY4741 derivate; *yrr1::loxP*; pJFE1-*YRR1* | This work |
| BY4741(*yrr1Δ+YRR1^T38A^*) | BY4741 derivate; *yrr1::loxP*; pJFE1- *YRR1^T38A^* | This work |
| BY4741(*yrr1Δ+YRR1^Y134A^*) | BY4741 derivate; *yrr1::loxP*; pJFE1- *YRR1^Y134A^* | This work |
| BY4741(*yrr1Δ+YRR1^S155A^*) | BY4741 derivate; *yrr1::loxP*; pJFE1- *YRR1^S155A^* | This work |
| BY4741(*yrr1Δ+YRR1^S176A^*) | BY4741 derivate; *yrr1::loxP*; pJFE1- *YRR1^S176A^* | This work |
| BY4741(*yrr1Δ+YRR1^T180A^*) | BY4741 derivate; *yrr1::loxP*; pJFE1- *YRR1^T180A^* | This work |
| BY4741(*yrr1Δ+YRR1^T185A^*) | BY4741 derivate; *yrr1::loxP*; pJFE1- *YRR1^T185A^* | This work |
| BY4741(*yrr1Δ+YRR1^S186A^*) | BY4741 derivate; *yrr1::loxP*; pJFE1- *YRR1^S186A^* | This work |
| BY4741(*yrr1Δ+YRR1^T610A^*) | BY4741 derivate; *yrr1::loxP*; pJFE1- *YRR1^T610A^* | This work |
| BY4741(*yrr1Δ+YRR1^S745A^*) | BY4741 derivate; *yrr1::loxP*; pJFE1- *YRR1^S745A^* | This work |
| BY4741(*yrr1Δ+YRR1^I756A^*) | BY4741 derivate; *yrr1::loxP*; pJFE1- *YRR1^I756A^* | This work |
| BY4741(*yrr1Δ+YRR1^I775A^*) | BY4741 derivate; *yrr1::loxP*; pJFE1- *YRR1^I775A^* | This work |
| BY4741(*yrr1Δ+YRR1^Y134E^*) | BY4741 derivate; *yrr1::loxP*; pJFE1- *YRR1^Y134E^* | This work |
| BY4741(*yrr1Δ+YRR1^T185E^*) | BY4741 derivate; *yrr1::loxP*; pJFE1- *YRR1^T185E^* | This work |
| BY4741(*yrr1Δ+YRR1^Y134A/T185A^*) | BY4741 derivate; *yrr1::loxP*; pJFE1- *YRR1^Y134A/T185A^* | This work |
| BY4741(*yrr1Δ+YRR1^Y134A^*-GFP) | BY4741 derivate; *yrr1::loxP*; pJFE3-*YRR1^Y134A^*-GFP | This work |
| BY4741(*yrr1Δ+YRR1^T185A^*-GFP) | BY4741 derivate; *yrr1::loxP*; pJFE3-*YRR1^T185A^*-GFP | This work |
| BY4741(*yrr1Δ+YRR1^Y134A/T185A^*-GFP) | BY4741 derivate; *yrr1::loxP*; pJFE3-*YRR1^Y134A/T185A^*-GFP | This work |
| CEN.PK2-1C | *MATa; ura3-52; trp1-289; leu2-3,112; his311; MAL2-8C; SUC2* | EUROSCARF |
| CEN.PK2-1C (*yrr1Δ*) | CEN.PK2-1C derivate; *yrr1::loxP* | This work |
| CEN.PK2-1C (pJFE1) | CEN.PK2-1C derivate; pJFE1 | This work |
| CEN.PK2-1C (*yrr1Δ+*pJFE1) | CEN.PK2-1C derivate; *yrr1::loxP*; pJFE1 | This work |
| CEN.PK2-1C (*yrr1Δ+YRR1^WT^*) | CEN.PK2-1C derivate; *yrr1::loxP*; pJFE1-*YRR1* | This work |
| CEN.PK2-1C (*yrr1Δ+YRR1^Y134A^*) | CEN.PK2-1C derivate; *yrr1::loxP*; pJFE1-*YRR1^Y134A^* | This work |
| CEN.PK2-1C (*yrr1Δ+YRR1^T185A^*) | CEN.PK2-1C derivate; *yrr1::loxP*; pJFE1-*YRR1^T185A^* | This work |

**Table S2.** List of primers used for plasmids and strain construction in this work

| Primer name | Sequence (5’-3’) |
| --- | --- |
| *MSN5-*KO-UP1 | ATGGATTCCACAGGCGCTTCTC |
| *MSN5-*KO-UP2 | GAAGTTATTAGGTGATATCAGATCCACTAGTGGCCTATGCAAAGGGCACTCATGATTGATTTTATAGATTGGG |
| *MSN5-*KO- DOWN1 | ATTAAGGGTTGTCGACCTGCAGCGTACGAAGCTTCAGCTGCTTCACTGCATGGTCTAGTAATCCTAGTCC |
| *MSN5-*KO- DOWN2 | TCAGTTGTCATCAAAGAGATTACCCACAGCAC |
| *MSN5-*KO-YZ | GGGGAAATTCATGCCCGCATTTTTG |
| *YRR1-*GFP-UP1 | CGCGGATCCATGAAAAGAAGAAGCGATGCTTTGTTGGG |
| *YRR1-*GFP-UP2 | TCTAAAGGTGAAGAATTATTCACTGGTGTTGTCCC |
| *YRR1-*GFP-DOWN1 | GGGACAACACCAGTGAATAATTCTTCACCTTTAGACATGGAACCACCACCATTGTCTTTGTAATCATCGAAAAGAGTGGAAAATTCAG |
| *YRR1-*GFP-DOWN2 | TGCACTGCAGTTATTTGTACAATTCATCCATACCATGGGTAATACCAGC |
| *SNG1*-QPCR-F | TGAGGAAGAGAAGGGCGGTA |
| *SNG1*-QPCR-R | ACAGGCACCCCAGTAAATCG |
| *SNQ2*-QPCR-F | GATGAGCAGAGCGATACCCC |
| *SNQ2*-QPCR-R | TTAAAGGCACCGCTTCTCGT |
| *YOR1*-QPCR-F | ACAAGATGAATGAGGCGGGA |
| *YOR1*-QPCR-R | AAAACACCAACTGAAGCCGC |
| Actin-F | CAAACCGCTGCTCAATCTTC |
| Actin-R | AGTTTGGTCAATACCGGCAG |
| *MSN5-*KO-UP1 | ATGGATTCCACAGGCGCTTCTC |
| *MSN5-*KO-UP2 | GAAGTTATTAGGTGATATCAGATCCACTAGTGGCCTATGCAAAGGGCACTCATGATTGATTTTATAGATTGGG |
| *MSN5-*KO- DOWN1 | ATTAAGGGTTGTCGACCTGCAGCGTACGAAGCTTCAGCTGCTTCACTGCATGGTCTAGTAATCCTAGTCC |
| *MSN5-*KO- DOWN2 | TCAGTTGTCATCAAAGAGATTACCCACAGCAC |
| *MSN5-*KO-YZ | GGGGAAATTCATGCCCGCATTTTTG |
| *YRR1-*GFP-UP1 | CGCGGATCCATGAAAAGAAGAAGCGATGCTTTGTTGGG |
| *YRR1-*GFP-UP2 | TCTAAAGGTGAAGAATTATTCACTGGTGTTGTCCC |
| *YRR1-*GFP-DOWN1 | GGGACAACACCAGTGAATAATTCTTCACCTTTAGACATGGAACCACCACCATTGTCTTTGTAATCATCGAAAAGAGTGGAAAATTCAG |
| *YRR1-*GFP-DOWN2 | TGCACTGCAGTTATTTGTACAATTCATCCATACCATGGGTAATACCAGC |
| *SNG1*-QPCR-F | TGAGGAAGAGAAGGGCGGTA |
| *SNG1*-QPCR-R | ACAGGCACCCCAGTAAATCG |
| *SNQ2*-QPCR-F | GATGAGCAGAGCGATACCCC |
| *SNQ2*-QPCR-R | TTAAAGGCACCGCTTCTCGT |
| *YOR1*-QPCR-F | ACAAGATGAATGAGGCGGGA |
| *YOR1*-QPCR-R | AAAACACCAACTGAAGCCGC |
| *YRR1*-UP | CGCGGATCCATGAAAAGAAGAAGCGATGCTTTGTTGG |
| *YRR1*-DOWN | CTGCAGTGCATTAATTGTCTTTGTAATCATCGAAAAGAGTGGAAAATTC |
| *YRR1-*mutant-F1 | CGCGGATCCGCGATGAAAAGAAGAAGCGATGC |
| *YRR1-*mutant-R2 | TGCACTGCAGTTAATTGTCTTTGTAATCATCGAAAAGAGTG |
| T38A-F1 | GTAACTCAGGAGCACCAACTTCTACTAGC |
| T38A-R1 | GCTAGTAGAAGTTGGTGCTCCTGAGTTAC |
| T134A-F1 | GCGAGCTCCATGGCAACTTCGCCAAATTTC |
| T134A-R1 | GAAATTTGGCGAAGTTGCCATGGAGCTCGC |
| S155A-F1 | CTACTGAAACTTCAGCACCATTACCCGATG |
| S155A-R1 | CACCATCGGGTAATGGTGCTGAAGTTTCAGTAG |
| G176A-F1 | CTACAAAGTAAACATGCAGGAAGATCAACACTATAC |
| G176A-R1 | GTATAGTGTTGATCTTCCTGCATGTTTACTTTGTAG |
| T180A-F1 | CATTCCGGAAGATCAGCACTATACGG |
| T180A-R2 | GCCGTATAGTGCTGATCTTCCGGAATG |
| T185A-F1 | CTATACGGCCCCGCATCTATGAGAACC |
| T185A-R1 | GGTTCTCATAGATGCGGGGCCGTATAGTG |
| S186A-F1 | CGGCCCCACTGCAATGAGAACCCA |
| S186A-R1 | TGGGTTCTCATTGCAGTGGGGCCG |
| T610A-F1 | GCTTTTTTCCAAAGCATTGGTGTTTTTTTGTTC |
| T610A-R1 | GAACAAAAAAACACCAATGCTTTGGAAAAAAGC |
| S745A-F1 | GCGGGATCTCGCACTTGCAGTTCCAACG |
| S745A-R1 | CGTTGGAACTGCAAGTGCGAGATCCCGC |
| I756A-F1 | CCCACGCCCTCAATAGCACCAATGTTACCCTC |
| I756A-R1 | GAGGGTAACATTGGTGCTATTGAGGGCGTGGG |
| I775A-F1 | AGAGTCAATCAGAGGCAATTCAGATGCTGACAGATGA |
| I775A-R1 | TCATCTGTCAGCATCTGAATTGCCTCTGATTGACTCT |
| T134E-F1 | GCGAGCTCCATGGAAACTTCGCCAAATTTC |
| T134E-R1 | GAAATTTGGCGAAGTTTCCATGGAGCTCGC |
| T185E-F1 | CTATACGGCCCCGAATCTATGAGAACC |
| T185E-R1 | GGTTCTCATAGATTCGGGGCCGTATAGTG |
| *YRR1-*GFP-UP1 | CGCGGATCCATGAAAAGAAGAAGCGATGCTTTGTTGGG |
| *YRR1-*GFP-UP2 | TCTAAAGGTGAAGAATTATTCACTGGTGTTGTCCC |
| *YRR1-*GFP-DOWN1 | GGGACAACACCAGTGAATAATTCTTCACCTTTAGACATGGAACCACCACCATTGTCTTTGTAATCATCGAAAAGAGTGGAAAATTCAG |
| *YRR1-*GFP-DOWN2 | TGCACTGCAGTTATTTGTACAATTCATCCATACCATGGGTAATACCAGC |

**Additional figures**


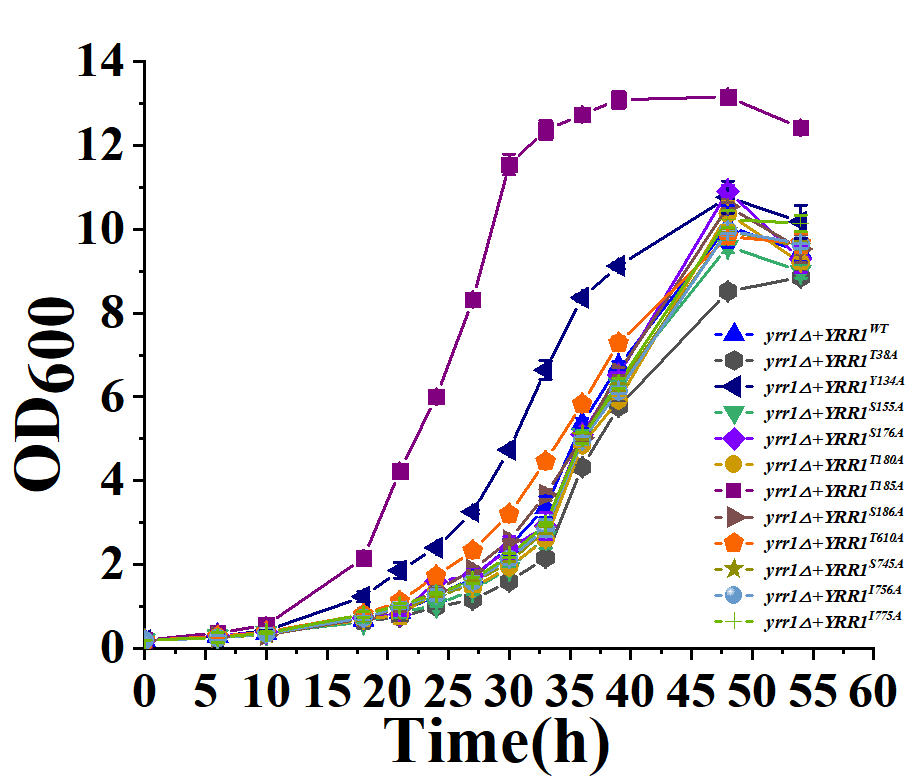


**Figure S1.** Growth curve of all eleven point mutants under 6 mM vanillin stress in SC-Ura medium. The error bar represents three times the standard deviation.


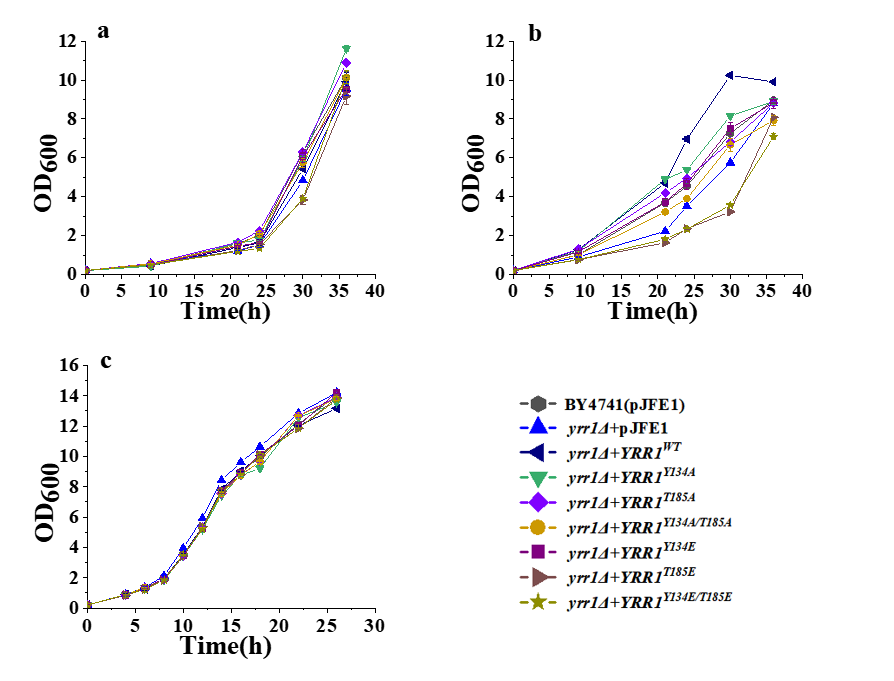


**Figure S2.** Resistance test of recombinant strains. The host strains were all BY4741. Incubate in SC-Ura liquid medium supplemented with 12 mM furfural (a), 20 mM HMF (b) and no inhibitor (c) at 30℃. The error bar represents three times the standard deviation.


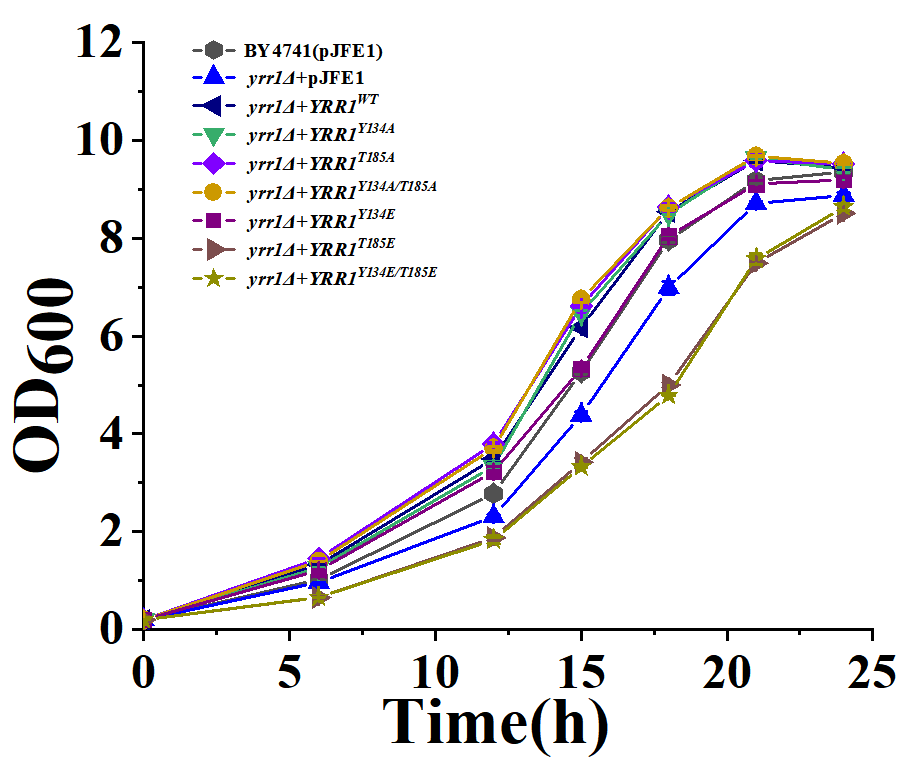


**Figure S3.** Resistance test of recombinant strains. The host strains were all BY4741. Incubate in SC-Ura liquid medium supplemented with 0.05 mg L^-1^ 4NQO at 30℃. The error bar represents three times the standard deviation.


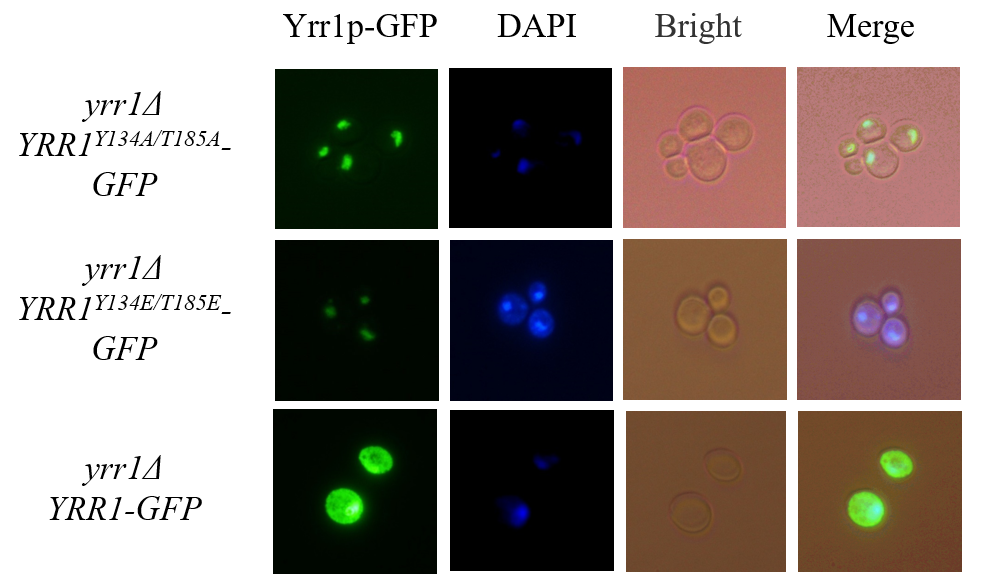


**Figure S4.** Subcellular localization of two site phosphorylation and dephosphorylation mutations. The samples were cultured in SC-Ura. Intracellular localization was analyzed by fluorescence microscope (green). Nuclear DNA was stained with DAPI (blue).


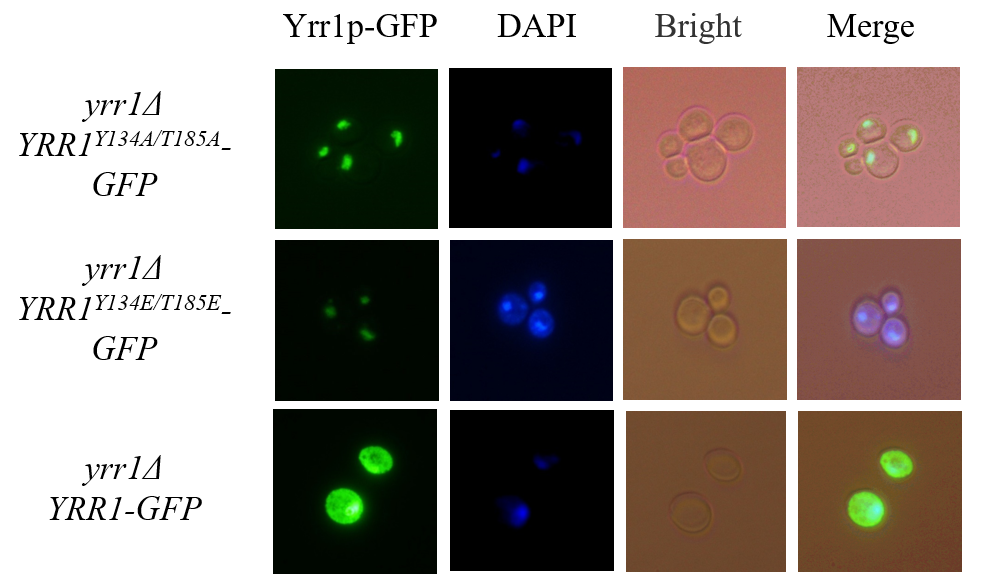


**Figure S5.** Subcellular localization of two site phosphorylation and dephosphorylation mutations. The samples were cultured in SC-Ura. Intracellular localization was analyzed by fluorescence microscope (green). Nuclear DNA was stained with DAPI (blue).
